# Disinhibited Revenge – An fNIRS Study on Forgiveness and Cognitive Control

**DOI:** 10.3389/fnbeh.2019.00223

**Published:** 2019-09-26

**Authors:** Moritz Julian Maier, David Rosenbaum, Florian Benedikt Haeussinger, Martin Brüne, Andreas Jochen Fallgatter, Ann-Christine Ehlis

**Affiliations:** ^1^Psychophysiology and Optical Imaging, Department of Psychiatry and Psychotherapy, University of Tuebingen, Tuebingen, Germany; ^2^Graduate School of Neural and Behavioral Sciences, University of Tuebingen, Tuebingen, Germany; ^3^Department of Psychiatry, Psychotherapy and Preventive Medicine, Division of Cognitive Neuropsychiatry and Psychiatric Preventive Medicine, LWL University Hospital Bochum, Ruhr-University Bochum, Bochum, Germany; ^4^LEAD Graduate School & Research Network, University of Tuebingen, Tuebingen, Germany; ^5^Werner Reichardt Centre for Integrative Neuroscience (CIN), Tuebingen, Germany

**Keywords:** cognitive control, forgiveness, fNIRS, revenge, impulsivity, dictator game, inhibition, emotion regulation

## Abstract

The ability to reconcile is a key factor for a cooperative and successful life. Among the many factors that have an impact on how people negotiate social contracts, poor cognitive control (which is inversely linked to impulsivity) may exert negative effects on forgiveness. To investigate the neurobiological basis of this proposition, subjects with high vs. low impulsivity scores completed an ultimatum game (UG) and a dictator game (DG). First, the participants played an UG where they had to accept or reject offers from fair or unfair opponents. Afterward, the roles changed, and a DG was played. Here, subjects had the opportunity to forgive or take revenge on unfair opponents by the allocation of a fair/unfair amount of money. During this task, activity of the dorsolateral prefrontal cortex (DLPFC) was assessed *via* functional near-infrared spectroscopy (fNIRS). Highly impulsive subjects were significantly more revenge-seeking than individuals with a low impulsivity. This behavioral difference was reflected in the activation pattern of the left DLPFC, where higher activation in trials with unfair opponents was found, but only in the highly impulsive group. This result is discussed as an indicator of more revenge-driven behavior in highly impulsive individuals, since activity in the left DLPFC is associated with retaliation.

## Introduction

“*The weak can never forgive. Forgiveness is an attribute of the strong*.”

Gandhi

Stability of social relations, academic success, potential conflicts with the law—all of these highly relevant factors for a desirable life are strongly correlated with the concept of cognitive control (e.g., Nota et al., 2004; Moffitt et al., 2011; Inzlicht et al., 2015). While persons with high cognitive control are successful in various areas of life, a lack of cognitive control is often associated with poor psychosocial functioning. Several neuropsychiatric disorders such as attention deficit hyperactivity disorder (ADHD; e.g., Barth et al., 2015), drug dependence (e.g., Verdejo-Garcıa et al., 2005; Barth et al., 2015) or Borderline Personality Disorder (Brüne et al., 2013) frequently display low states of cognitive control. Moreover, rumination—which is strongly connected to depression—is associated with a lack of cognitive control (Rosenbaum et al., 2017, 2018c), while conversely, therapeutic approaches to reduce rumination aim at improving cognitive control (Rosenbaum et al., 2018b). Cognitive control mainly consists of three neuropsychological subfunctions; updating, shifting, and inhibition (Miyake et al., 2000). Accordingly, updating can be seen as the persistent monitoring and task-based removal/adding of relevant content, while shifting describes flexible moving between different tasks or mental states; inhibition is defined as the suppression of prepotent (but not goal-oriented) response tendencies (Miyake and Friedman, 2012). Generally, cognitive control is negatively correlated with impulsivity (e.g., Bari and Robbins, 2013).

Cognitive control plays a central role in the negotiation of social contracts of all kinds, including deception and reconciliation (Karremans and van der Wal, 2013). The importance of successful forgiveness, for example, is underlined by associations with general health outcomes [and especially cardiovascular health (Friedberg et al., 2007)], stress perception (Worthington et al., 2007) and overall mortality (Toussaint et al., 2012). Forgiveness can be described as a fluent process which consists of two steps; first, the decision to forgive the provocateur, and second, the inhibition of revenge-seeking feelings (Fincham et al., 2006; Wilkowski et al., 2010). These feelings, like anger and hate, are a natural reflexive response to transgressions, according to Pingleton (1989). Therefore, inhibition in particular (as a subfunction of cognitive control) is discussed as a key factor in successful reconciliation. Neurobiologically, the conflict monitoring theory (Botvinick et al., 2001), posits that potential response conflicts (e.g., the decision to forgive vs. the impulsive desire for revenge) are associated with the activation of the anterior cingulate cortex (ACC), which signals an increased need for the implementation of cognitive control to the dorsolateral prefrontal cortex (DLPFC; e.g., Kerns et al., 2004; Egner and Hirsch, 2005). According to this conceptual embedding, differences between high- and low forgiving individuals should be visible, especially in the DLPFC.

However, it should be noted that besides the DLPFC, other brain areas are also involved in forgiveness. For example, Ricciardi et al. (2013) found significant covariations between the ACC, the DLPFC and the inferior frontal gyrus (IFG) during forgiveness processes. According to the authors, the ACC is associated with affective and emotional processing in forgiveness (Bush et al., 2000), while the IFG is associated with cognitive and emotional empathy (Shamay-Tsoory et al., 2009). Although other brain regions are important for a complex cognitive process such as forgiveness, the DLPFC has been selected as the area of interest in this study, as it is thought that this brain region controls areas such as the IFG and ACC during forgiveness processes (Clark, 2005).

To study the neurobiological basis of revenge and forgiveness, Brüne et al. (2013) developed a study design, which enabled participants to forgive unfair opponents or to take revenge in a controllable experimental setting. To this end, the participants first played an ultimatum game (UG) where a virtual opponent split up 10 Euro on each trial and the participants had to accept or reject the offer. During the game, the participants learned implicitly that half of the opponents were fair (offers between 3 and 5 Euro) and the other half were unfair (offers between 0 and 2 Euro). Subsequently, the roles changed, and the subjects had to split up 10 Euro between themselves and the previous opponents in a dictator game (DG). Here, subjects had the possibility to forgive their previously unfair opponents or to take revenge. The slight difference between the UG and the DG, when considering the rejection possibilities is important to note: since the opponents had no possibility to reject an offer made by the participants in the DG, the subjects were able to allocate the money without any fear of rejection. In their study, Brüne et al. (2013) found a significantly higher activation of the right DLPFC when subjects “forgave” their previously unfair opponents (by allocating a fair amount of money themselves) in comparison to allocating a fair amount of money to a previously fair opponent. This result can be interpreted as an indicator that forgiveness processes are (partly) controlled by the DLPFC and thus by a classical cognitive control region. To further assess the causality of this finding, Maier et al. (2018) combined the paradigm of Brüne et al. (2013) with an inhibitory continuous theta-burst stimulation (cTBS, Huang et al., 2005) in order to test the effects of reduced activity in the right DLPFC in forgiveness behavior. In this study, reduced forgiveness (i.e., more revenge-seeking) behavior towards previously unfair opponents was found, after inhibition of the right DLPFC *via* the cTBS. The emotions experienced towards the opponents were the same in both conditions. Strong negative emotions towards unfair opponents and positive emotions towards fair opponents. Along similar lines, in a study, Müller-Leinß et al. (2018) found that when using repetitive transcranial magnetic stimulation (rTMS), inhibition of the right DLPFC not only led to an increased punishment of previously unfair opponents, but also to less fair behavior toward previously fair players, suggesting maximization of one’s own monetary benefit in a “homo economicus”-like fashion.

To further investigate the connection between cognitive control and forgiveness behavior, the question arises how forgiveness behavior differs between subjects with high- vs. low cognitive control. If subjects with low cognitive control would act in a less forgiving manner, it would indicate that this subgroup fails in inhibiting revenge-seeking feelings. To clarify this potential correlation, we compared subjects with high vs. low cognitive control (as defined by low vs. high impulsivity scores) with the combination of an UG and a DG. To control the cognitive control abilities of the subjects in an objective and reliable way, an Emotional Stroop-task was used to assess both cognitive control and implicit emotions. In the Emotional-Stroop task, color words of the classical Stroop-task are replaced with emotional vs. non-emotional words. Using this task, which was run after the UG and DG, it was possible to measure both the cognitive control and the implicit emotionality of the participants. Based on previous work using this paradigm (Brüne et al., 2013; Müller-Leinß et al., 2018; Maier et al., 2018) and the outlined theoretical considerations, we propose the following hypotheses: subjects with low cognitive control will allocate unfair amounts of money to unfair opponents more often than subjects with high cognitive control (i.e., more impulsive retaliation). We expect no differences between groups towards the fair opponents because the interaction lacks the provocation of revenge. These specific effects should be accompanied by activation differences in the right DLPFC: we expect significantly less activation in the right DLPFC in subjects with low cognitive control compared to subjects with high cognitive control. This difference between the groups should be particularly accentuated in trials where the subjects face previously unfair opponents, due to a high need for cognitive control in terms of the inhibition of revenge-seeking behavior.

## Materials and Methods

### Subjects

Subjects with high- vs. low cognitive control were screened *via* online questionnaires to assess demographic data, potential exclusion criteria, and impulsivity scores using the impulsivity scale of the adult ADHD self-report scale (ASRS; Kessler et al., 2005). Exclusion criteria included chronic or acute diseases that can influence the cerebral metabolism (moderate or severe craniocerebral trauma, kidney insufficiency, diabetes and unattended hypertension) or acute endangerment of the self or others. Additionally, they were asked if they were at present or in the past under medical treatment because of neurological or psychiatric illness or if they took any (illegal) drugs the last month. In case of uncertainty regarding this question there was a free-text field where potential subjects were able to indicate potential problems. Subjects with scores between 15 and 23 on the ASRS were assigned to the high impulsivity group (=low cognitive control); subjects with scores lower than 10 were assigned to the low impulsivity group (=high cognitive control). These thresholds were already used in various previous studies (e.g., Herrmann et al., 2009) and provided clearly differentiable participant groups without recruiting a clinical group. This study was approved by the ethics committee of the Medical Faculty of the University of Tübingen, in accordance with the current version of the Declaration of Helsinki. Written informed consent was obtained from all participants.

In total, 67 subjects participated. Twenty-nine were assigned to the low impulsivity subject group, 38 to the highly impulsive subject group. The mean age was 34.4 years (SD = 2.95), 50 participants were females, 17 males. Considering age and sex no significant differences were observed (*t*_(65)_ = 1.11, *p* = 0.271; *χ*^2^ = 0.592, *p* = 0.442).

### Experimental Process

After arriving and signing the written informed consent form, the functional near-infrared spectroscopy (fNIRS) probeset was mounted and the experiment started with the UG, which was directly followed by the DG. Other tasks, which were part of a different study and are reported elsewhere, followed approximately 30 min after the DG. At the end, an Emotional Stroop task (Williams et al., 1996) was run to further assess cognitive control capacities as well as emotionality.

### Paradigm

The paradigm was adapted from Brüne et al. (2013) and consisted of two subsequent tasks, an UG followed by a DG. Every game consisted of 40 trials in total and had a duration of approximately 9 min. First, an UG was played against four virtual opponents. During each trial, the opponent split up 10 Euro (virtual money, 10 trials per opponent, randomized order) between themselves and the subject. The participants had the choice to accept or to reject the offer. In case of a rejection, neither the subject nor the opponent received any money. Therefore, a rejection was also an option to punish unfair offers made by the opponents. During this task, the subjects implicitly learned that there are two fair (one male, one female; offers between 3 and 5 Euro) and two unfair opponents (offers between 0 and 2 Euro). The classification of fair and unfair offers was made based on previous studies (e.g., Sanfey et al., 2003; Brüne et al., 2013). Every trial began with the presentation of the name and face of the opponent for 3 s, which was followed by a jittered 2–3 s anticipation period. After that, subjects were presented with the offer of the opponent for 3 s. During this decision period, subjects had to indicate their response (acceptance vs. rejection) *via* a button press. After that, a feedback screen was presented for 3 s. An inter-trial interval of jittered 2–3 s followed subsequently.

After the completion of the UG, a DG was played. Here, the roles changed, and the participants had to split up the money. The opponents (now the recipients) were the persons introduced in the previous UG. As in the UG, 40 trials—10 per opponent—were played. An important difference in comparison to the previously played UG is that the opponents had no possibility to reject the offers made by the participants (which clearly reduces the fear of punishment for unfair money allocations). The timing and order were (beside the no choice circumstance) the same as in the UG. In both games, the participants had the instruction to imagine that they were playing for real money and with real persons (with the aim to increase the involvement of the participants). As we used computer opponents with pictures taken from the study of Brüne et al. (2013), the participants were not familiar with the four different characters of the game before the ultimatum- and DG. In both paradigms, the participants were seated in front of an Eizo^®^ 22-inch screen, at a distance of approximately 60 cm. Only participants with normal or corrected visual capabilities were included.

### Emotional Stroop Task

Cognitive control and affective state were measured with an Emotional Stroop task (Watts et al., 1986). Based on the stimuli of Smith and Waterman (2003), the task consisted of negative, positive and neutral words (10 stimuli per category). These 30 words were presented in four different colors (blue, green, red, yellow), resulting in 120 different stimuli, which were presented in the center of a black screen. The responses were assessed *via* a button box with one button per color. As a reminder, a button-color-assignment was presented during the whole experiment. In the beginning, 20 training trials with a correct/incorrect feedback were run. Subsequently, the experiment started with a fixation cross for 200 ms, followed by a target stimulus until response (timeout after 1,000 ms). In the experimental trials, no feedback was presented. Between the trials, a jittered break of 4,000–7,000 ms appeared (Plichta et al., 2007).

### fNIRS

To assess cortical activation of the DLPFC during the DG, fNIRS was used. Biological tissue (e.g., skin or bones) is relatively transparent for near-infrared light, and oxygenated (O_2_Hb) and deoxygenated (HHb) hemoglobin absorb near-infrared light with different absorption spectra (Fallgatter et al., 2004; Haeussinger et al., 2011). Due to these preconditions, it is possible to measure relative changes in O_2_Hb and HHb in the upper 2–3 cm of the cortex. Based on the principle of neurovascular coupling, a decrease of HHb and an increase in the concentration of O_2_Hb indicates cortical activation within a specific brain region. The measurements for this study were run using a commercial multi-channel fNIRS system (ETG-4000 Optical Topography System; Hitachi Medical Company, Japan) with a temporal resolution of 10 Hz. A 3 × 11 probeset with 52 channels (16 detectors, 17 emitters, and an interoptode at distance of 3 cm) was oriented on a reference point Fpz and T3/T4 based on the international 10-20 system (Jasper, 1958).

### Questionnaires

In addition to the questionnaires for the screening of suitable participants, forgiveness and cognitive control-related variables were assessed. These questionnaires were completed online *via* a Sosci Survey (Leiner, 2018) within 1 week before the measurement. The following questionnaires were used: the Beck Depression Inventory (BDI; Beck et al., 1996), the Tendency to Forgiveness Scale (Brown, 2003) and the Willingness to Forgive Scale (Allemand et al., 2008). After the experiment, the desire for revenge and sympathy perception (0–5, 0 = low feelings of sympathy/revenge) of the participants towards their opponents was additionally assessed.

### Statistical Processing (Behavioral Data)

The rejection rate in the UG between both groups was compared with an unpaired *t*-test. To test the hypothesis of an interaction effect of fairness of the opponent (fair vs. unfair) and group of the subject (high vs. low cognitive control), a non-parametrical permutation test was used for the analysis of money allocation during the DG, due to non-normally distributed data. First, using a permutation test for repeated measurements, the differences between the offers towards fair vs. unfair opponents were analyzed. Second, the difference between offers_(towards fair opponents)_ and offers_(towards fair opponents)_ was compared between the groups by a comparison of difference scores (Δfair Opponent − unfair Opponent) using the same test method (see e.g., Gibbons and Chakraborti, 2011). For all analyses, MATLAB 2015b (The MathWorks, Natick, MA, USA) or SPSS 22 (SPSS Inc., Chicago, IL, USA) were used.

### Statistical Processing (fNIRS Data)

**Figure 1:**
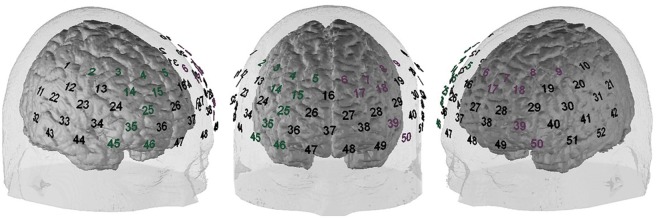
Functional near-infrared spectroscopy (fNIRS) probeset position. Green numbers indicate the right dorsolateral prefrontal cortex (DLPFC), pink numbers indicate the left DLPFC.

All fNIRS data were exported without any pre-processing. For all following analyses, MATLAB 2017 (The MathWorks, Natick, MA, USA) was used. All frequencies <0.01 Hz and >0.5 Hz were excluded with a bandpass filter. For the correction of motion artifacts, the correlation based signal improvement (cbsi) procedure of Cui et al. (2010) was used, and the resultant cbsi-hb was used for all subsequent analyses. Additionally, an Independent Component Analysis (ICA; Delorme and Makeig, 2004) was applied to exclude high amplitude artifacts. The left and the right DLPFC were defined as regions of interest (ROIs); the allocation of NIRS channels to these ROIs was made in accordance with Rorden and Brett (2000), Singh et al. (2005) and Tsuzuki et al. (2007). The positions of the ROIs are depicted in Figure 1. Afterward, the mean activation of the ROIs was extracted for further analyses. First, a 2 × 2 ANOVA with the within-subjects factor opponent (fair vs. unfair) and the between-subjects factor group (highly impulsive vs. low impulsivity) was run, separately for each ROI. As *post hoc* tests, *t*-tests were used. For a better comparability, the fNIRS data was *z* transformed. The factor of money allocation was not included because of different frequencies in the different conditions/groups. For example, the combination “unfair offer towards a previously fair opponent” was absent. Especially in the response to unfair opponents, the frequency of fair vs. unfair offers was so different between the groups that a comparison of the fNIRS data did not seem to make much sense. Additionally, with the combined analysis of all trials (independent of the exact money allocation), statistical power was increased, and we were able to investigate the mechanisms underlying behavioral differences between the groups.

### Statistical Processing (Stroop Data)

For analysis of the Stroop data, the inversed efficiency score ($IES=\frac{RT}{1-\text{Proporation of errors}}$; Townsend and Ashby, 1983) was used. Trials with differences of more than two standard deviations from the mean per person (in total 3.72%) and incorrect trials were excluded from the analyses. After an ANOVA where no effect for stimulus valence was found, the data were merged for valence and the difference between the groups was assessed with a *t*-test for independent measurements.

### Statistical Processing (Correlations)

To analyze potential brain-behavior correlations, the frequency of fair responses towards unfair opponents was calculated (=forgiveness behavior). Subsequently, this frequency was correlated (Pearson method) to the event related average (ERA) of the left DLPFC (referring to the fNIRS results) for the trials with fair and unfair opponents. The *α*-value was adjusted for multiple tests using the Bonferroni method (Dunnett, 1955).

### Statistical Processing (Logistic Regression Analysis)

To further analyze the results, a logistic regression separated for the groups (low vs. highly impulsive) was run. The dependent variable was the number of trials with fair offers [fair offers were defined as offer ≥3 € (see Brüne et al., 2013)] towards unfair opponents (=frequency of forgiveness); independent variables were the activation in the right and left DLPFC in trials with unfair opponents, the IES, the scores of the Tendency to Forgiveness Scale, and the Willingness to forgive Scale as well as the scores of revenge and sympathy feelings of participants towards unfair opponents.

## Results

### Stroop Task and DG Behavioral Results

In line with our hypothesis, the highly impulsive subject group had a significantly higher IES score (indicative of lower cognitive control) than the low impulsivity subject group (*t*_(53)_ = −2.53, *p* = 0.014; M_highly impulsive_ = 724.08 vs. M_low impulsivity_ = 650.89).

**Figure 2:**
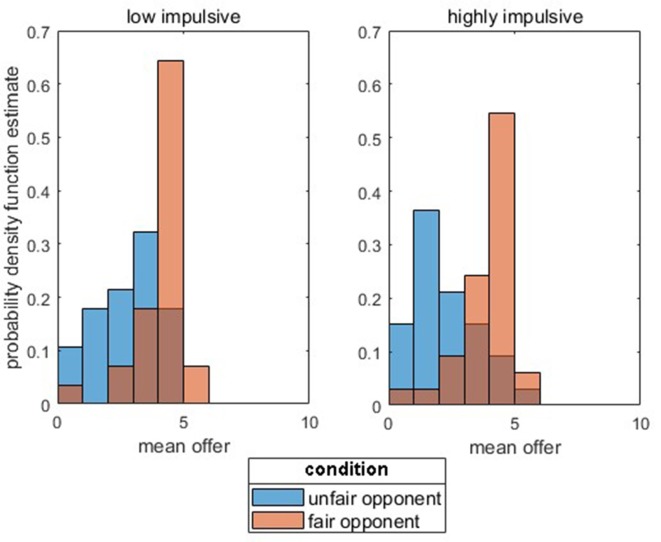
Probability function estimate for low impulsivity vs. highly impulsive subjects for the mean offers (in €) separated for unfair (blue columns) vs. fair (orange columns) opponents.

The rejection rate in the UG did not significantly differ between the groups (*t*_(53)_ = 1.01, *p* = 0.315). As expected, for behavior in the DG (mean amount of allocated money) towards previously fair opponents, no effect was found [*p* > 0.05; Mean_low_impulsive_ = 4.08 € (*SD* = 0.96), Mean_highly_impulsive_ = 3.86 € (*SD* = 0.99)]. For unfair opponents, a significant difference between the groups was found [*p* < 0.05; Mean_low_impulsive_ = 2.86 € (*SD* = 1.21), Mean_highly_impulsive_ = 2.20 € (*SD* = 1.24)]. Permutation tests for the double contrast (highly impulsive vs. low impulsivity for fair vs. unfair opponents) further indicate a significant interaction between both factors [group × opponent; *p* < 0.05, Δ_low_impulsivity (fair Opponent − unfair Opponent)_ = 1.22 € (*SD* = 0.88) vs. Δ_highly_impulsive (fair Opponent − unfair Opponent)_ = 1.65 € (*SD* = 0.97)]. Figure 2 depicts the probability density function estimate separately for the group and opponents.

### Questionnaire Results

For the results of the questionnaires and group comparisons, see Table 1. In a *t*-test for unrelated measurements, a significantly higher mean BDI was found in the highly impulsive group. Additionally, a significantly higher desire for revenge on unfair opponents was found in this group. In the low impulsivity group a marginally higher feeling of sympathy was found. The difference of the sympathy and desire for revenge experienced towards fair vs. unfair opponents differed significantly for both groups (in each case *p* < 0.001).

**Table 1:** Results of the different questionnaires separated by groups.

Questionnaire	Low impulsivity group (23 females, 6 males; *M*, *SD*)	Highly impulsive group (27 females, 11 males; *M*, *SD*)	*t*-value, *p*-value (one-tailed)
BDI	4.44, 3.13	9.60, 7.07	−4.00, <0.001*
Tendency to forgiveness Scale	15.25, 4.15	14.48, 4.67	0.68, 0.245
Willingness to forgive Scale	20.96, 5.12	21.21, 5.04	−0.19, 0.423
Desire for revenge (towards unfair opponents)	2.67, 0.99	3.13, 1.03	−1.79, 0.035*
Feelings of sympathy (towards unfair opponents)	2.10, 0.53	1.90, 0.59	1.43, 0.075

*Significant group difference (*p* < 0.05).

### fNIRS Results

**Figure 3:**
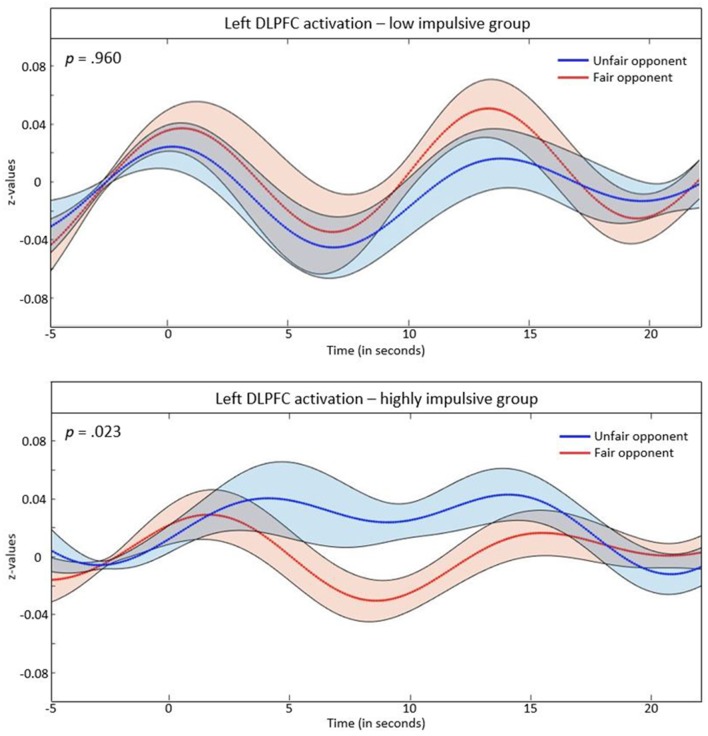
*z*-values of the event related average (ERA) signal in the left DLPFC separated for the low impulsivity and highly impulsive group. The shaded parts indicate the standard error of the mean.

We ran a 2 × 2 ANOVA with the within-subjects factor opponent (fair vs. unfair) and the between-subjects factor group (highly impulsive vs. low impulsivity) separated for the left and the right DLPFC. We found no effect in the right DLPFC. In the left DLPFC, a main effect for opponent (*F*_(1,65)_ = 4.53, *p* = 0.037) and an interaction effect of group and opponent was found (*F*_(1,65)_ = 4.28, *p* = 0.042). Subsequently, a *post hoc* *t*-test for repeated measurements was run separately for groups. No significant differences between fair and unfair opponents occurred in the low-impulsivity group (*t*_(37)_ = 0.51, *p* = 0.960). However, in highly-impulsive subjects, a significant difference between trials with fair vs. unfair opponents was found (*t*_(28)_ = 2.40, *p* = 0.023), with higher hemodynamic responses in the left DLPFC during money allocation to unfair opponents. These effects are depicted in Figure 3.

### Correlational Results

In the highly impulsive group, no significant correlations were found. In contrast, the low impulsivity group showed a significant negative correlation (*r* = −0.499, *p* = 0.018) between activation in the left DLPFC and the frequency of fair offers towards unfair opponents (=forgiveness behavior).

### Regression Analyses

For the low impulsivity group, only the perceived sympathy of the unfair opponent had a significant influence on the response towards unfair opponents (*F*_(1,22)_ = 7.36, *p* = 0.013, *n* = 23). With one point more in the sympathy rating (sympathy feelings towards unfair opponents), the low impulsivity subjects allocated on average 1.068 € more to unfair opponents. For the highly impulsive subjects, only the perceived revenge feeling towards unfair opponents had a significant influence on their money allocation towards unfair opponents (*F*_(1,21)_ = 6.85, *p* = 0.016, *n* = 22). With one point more in the revenge rating (revenge feelings towards the unfair opponents), the highly impulsive subjects allocated on average 4.096 € less to unfair opponents.

## Discussion

This study investigated the effects of cognitive control mechanisms on forgiveness towards unfair opponents in a combined ultimatum/DG. The results of the Emotional Stroop task confirmed the expected lower cognitive control capacity of the highly impulsive group. A significant difference between the groups was observed towards previously unfair opponents (where forgiveness is necessary for a fair response). As hypothesized, the highly impulsive group showed significantly less forgiveness/more revenge behavior. According to our hypotheses, we found no behavioral differences between groups towards previously fair opponents (control condition).

We also hypothesized that higher rates of forgiveness in the low impulsivity group would be accompanied with higher activity in the right DLPFC, comparable to other results in this field (e.g., Brüne et al., 2013; Maier et al., 2018). Surprisingly, we found no activation differences between fair and unfair opponents in the low impulsivity group and no group difference regarding the activation of the right DLPFC. In the left DLPFC, highly impulsive subjects exhibited significantly higher activation when playing against unfair opponents as compared to fair opponents. As it is assumed that cognitive control is needed to forgive (e.g., Pronk et al., 2010), and the left DLPFC is generally seen as a cognitive control region (e.g., Botvinick et al., 2001; Egner and Hirsch, 2005), this finding only in the highly impulsive group (which was less forgiving) is unexpected. To further analyze these unforeseen results, we ran a multiple regression analysis to explore the mechanisms underlying the different behavioral patterns in the low vs. highly impulsive group. While in subjects with low impulsivity only perceived sympathy for their virtual (unfair) opponents predicted money allocation, in the highly impulsive group revenge feelings significantly predicted the behavior. One explanation for the increased activation in the left DLPFC in highly impulsive subjects during money allocation to unfair opponents, might therefore lie within this revenge motivation. In a study of Strobel et al. (2011), higher activation in the left DLPFC was observed during a DG with the option for punishment. In line with this, Ricciardi et al. (2013) found higher left DLPFC activation during revenge in comparison to forgiving during social scenario evaluations. The stronger revenge-driven behavior of the highly impulsive subject group lines up very well with the results of Jones and Paulhus (2011) who also found more pronounced psychopathy and narcissism scores in persons with high impulsivity scores.

The fact that the low impulsivity group unexpectedly did not show increased activation in cognitive control areas, despite displaying more pronounced forgiveness behavior, might be explained by the specificities of the low impulsivity control group. It is assumed that cognitive control is needed to forgive due to the necessary suppression of unwanted (e.g., revenge-seeking) emotional feelings (Wilkowski et al., 2010; Maier et al., 2018). James and Taylor (2007) found that impulsivity is positively correlated with negative emotionality. This aligns well with the significantly lower desire for revenge in the low impulsivity group also after unfair treatment, which may have led to a reduced need to suppress unwanted revenge-seeking feelings *via* mechanisms of cognitive control. To summarize, the unexpected lack of significant activation in cognitive control areas (i.e., DLPFC) in the low impulsivity group could be explained by the fact that these subjects did not have any unwanted emotions to suppress, whereas the highly impulsive subjects were primarily revenge-driven in their behavior.

Alternatively, the unfair behavior of the highly impulsive group could also be interpreted as a more controlled and economically elaborated behavior, since allocating a small (“unfair”) amount of money makes sense from an economical perspective (e.g., Fehr and Fischbacher, 2004), depending on one’s motivational attitude. The higher activation in the left DLPFC as part of the cognitive control network could reflect this elaborated and cognitively controlled behavior. However, this interpretation would be contradictory to the results of the Emotional Stroop task and previous findings on the connection between impulsivity and (low) cognitive control (e.g., Fallgatter et al., 2005; Ehlis et al., 2008; Herrmann et al., 2010) and is therefore rather implausible.

Attention should also be given to the fact that the highly impulsive group indicated significantly higher values in the BDI. All subjects were far away from a pathological threshold (only subjects without psychiatric disorders were invited), nevertheless in the literature depression is linked with lower abilities to forgive (Tse and Cheng, 2006; Hirsch et al., 2011). But keeping the ecological validity in mind and the strong connection between the concepts of impulsivity and depression, an avoidance of these differences would not be useful. Furthermore, attention should be given for a potential influence of impulsivity on the behavior in the UG. In the present study, this was not the case but in other previous studies, the possibility of an influence was shown (see Crockett et al., 2010; Espín et al., 2015).

In future studies other brain regions like the posterior parietal cortex should also be studied, as this brain region, in combination with the DLPFC, is known to be part of the central executive network (Sridharan et al., 2008; Rosenbaum et al., 2018a). This network is *inter alia* responsible for social cognition which plays a crucial role in forgiveness processes (Sherman et al., 2014). More knowledge about the underlying network mechanisms would help in understanding the neural foundations of forgiveness processes to a new extent. Furthermore, other brain areas like the ACC and the IFG, which are known to play a role in forgiveness processes, could be investigated for their role in prosocial behavior in future studies. The highly significant differences in the sympathy and desire for revenge experienced towards fair vs. unfair opponents indicate that the manipulation used in the present study worked as planned. Nevertheless, in future studies a design in which participants are playing against real opponents while receiving a financial compensation based on their behavior during the game, could increase the personal involvement of the participants and could strongly influence the results, especially as there are previous works showing that there are differences in behavior between hypothetical and real scenarios (e.g., Clot et al., 2018; Ferguson et al., 2019).

In the present study, there was an imbalance between male and female participants. This difference was caused by the difficulty to recruit the same number of male and female participants who met the very specific inclusion criteria. Nevertheless, it is known that gender can have an influence on forgiveness processes, as woman are known to show more forgiveness behavior than men (Shackelford et al., 2002; Wade and Goldman, 2006). For further investigation of the neural foundations of these differences in future studies, researchers should aim for a gender balance.

Another potentially critical point of the present study is the various approaches used to analyze the results of the behavior in the DG and the Emotional Stroop task and the neural activation differences between the groups. Due to the different research questions targeted in this study with different tasks and approaches it was not possible to limit the statistical analyses to one specific test. Therefore, keeping a potential power inflection in mind, the results have to be interpreted with some caution, even if the discussed results seem robust. Additionally, the Emotional Stroop task was used after the UG/DG, and due to this order, it was possible to investigate the emotional influence of the gaming paradigms on different participant groups. It is potentially critical that due to this order there was a systematical influence of the gaming paradigms on the results of the Emotional Stroop task.

In conclusion, the results of this study provide new insights into the impact of impulsivity on forgiveness behavior and the underlying mechanisms of cognitive control. First, behavioral data indicate a difference in the ability and/or willingness to forgive between low impulsivity vs. highly impulsive subjects. Second, regression analyses and the fNIRS data indicate that these differences in retaliation are possibly based on different motivations: while the behavior of the low impulsivity group could mainly be associated with sympathy, the behavior of highly impulsive subjects might have been determined by feelings of revenge. Keeping the fundamental importance of reconciliation for health (Friedberg et al., 2007), coping with stress (Worthington et al., 2007) and overall mortality (Toussaint et al., 2012) in mind, the data in this study provide relevant insights into mechanisms underlying reduced forgiveness behavior in highly-impulsive subjects, with possible clinical implications, for example, for patients with ADHD, addiction or personality disorders.

## Data Availability Statement

The datasets generated for this study are available on request to the corresponding author.

## Ethics Statement

This study was approved by the ethics committee of the Medical Faculty of the University of Tübingen and was in accordance with the current version of the Declaration of Helsinki. Written informed consent was obtained from all participants.

## Author Contributions

MM, A-CE, FH, and MB contributed to the conception and design of the study. MM ran the study and organized the data. MM, DR, and A-CE performed the statistical analysis. MM wrote the first draft of the manuscript. A-CE, DR, and MB wrote sections of the manuscript. All authors critically revised the manuscript for important intellectual content and read and approved the submitted version.

## Conflict of Interest

The authors declare that the research was conducted in the absence of any commercial or financial relationships that could be construed as a potential conflict of interest.
